# Transparency in Hospital Medicine Metrics: An Effective Approach to Hospital Medicine Practice Management and Influencing Positive Behavior for Everyone's Benefit

**DOI:** 10.31486/toj.23.0020

**Published:** 2023

**Authors:** Michael R. Sewell

**Affiliations:** Department of Hospital Medicine, Louisiana State University Health–Shreveport, Shreveport, LA

Medicine is a competitive field, and internists are prone to being detail-oriented and outcomes-driven. The success or failure of hospital medicine programs is measured not only by outcomes (mortality, 30-day readmit rates) but also by quality metrics (case mix index, Hospital Consumer Assessment of Healthcare Providers and Systems [HCAHPS] scores). These metrics are increasingly becoming a marker for successful hospitalists. Just as an internist reviews test results, medical directors and practice managers are increasingly using metrics to find ways to improve the effectiveness and efficiency of their programs.

Hospitalists and their leadership teams should constantly pursue effective ways to optimize the metrics, patient outcomes, and fiscal success of their programs. Many programs have turned to financial incentives, and these can be quite successful. Pay-for-performance models and quality pay are common and can be an effective way for health systems and providers to work together toward common goals and patient outcomes.^1^

While financial incentives might be useful, money is not the only driver of physician performance. The competitive nature of physicians, their attention to detail, and their desire for positive outcomes are other possible motivating factors. But another tool can be very effective in improving metrics and achieving goals and outcomes in hospital medicine: transparency.

The call for transparency in health care is pervasive today, from patients, government, insurance companies, and patient advocacy groups. These stakeholders want openness with regard to data and pricing,^2^ and laws have been passed mandating transparency in certain aspects of health care.^3^ This type of transparency principally has to do with health care costs and performance to try to demonstrate value. In health care, value = (quality + outcomes) / cost.^4^

Transparency in health care also engenders trust. When processes are open for all to see, there are no surprises and fewer hidden agendas, and trust begins to grow. When everyone is held accountable and transparency exists, competition increases but outcomes improve as well. The patients get better care. The providers perform at a higher level. The institution's reputation improves and grows. In our experience, transparency in performance metrics—everyone sees everyone's numbers—leads to improvement in scores on these metrics and presumably in the outcomes that they represent.

With transparency already a factor in health care that has proven beneficial, then perhaps it remains an underutilized modality for effecting a desired result. Almost all hospitalist groups track metrics of all shapes and sizes and then report to each provider how they compare to the group and how they can improve. But many groups continue to blind the data, so that each provider only sees their own performance information. Transparency has a role here and is a powerful motivator of behavior in many positive ways.

When I was working at a large private health system and part of physician leadership there, we had a problem. A few of our providers just refused to do history and physical examinations (H&Ps). And unbelievably, we discovered that they were within their rights to do so according to our medical staff bylaws. Physicians actually had 30 days from the date of discharge to complete the medical record, and they took their time. Their action impaired patient care and billing/collection. In response, we changed the bylaws to penalize them with progressively larger fines until they were eventually paying $500 per month each for the convenience of doing H&Ps whenever they wanted—on their time. But the fine didn’t work, and the physicians continued to delay the completion of records.

Money alone was ineffective in changing physician behavior in that scenario. Ultimately, what motivated them to change their behavior was transparency. We projected a list of the physicians with delinquent H&Ps on our large screen during each department meeting and medical staff meeting. Through transparency, when all was known by all, the negative behavior stopped, and the process flourished.

There are many reasons why transparency may be absent in some hospitalist practices. Maybe leadership does not want to embarrass anyone or is afraid to hurt someone's feelings. But all practices are trying to achieve outstanding performance. We need to reward those who are setting the bar while simultaneously encouraging others to join them there. We should also be trying to encourage collegiality in achieving success as a team.

Transparency in the management of hospitalist practice may not be for everyone, but it has been hugely successful for us. We began an academic hospitalist program at a 300+ bed tertiary referral center in 2019. Our program began with 1 provider and we have grown to more than 30 hospitalists. We began measuring metrics immediately and initially struggled with the same issues many programs do: attribution, provider buy-in, communication, and shared goals with administration. We initially had very poor metric scores and limited success with improving them.

Then something changed. We needed to work on discharge before 10:00 am to align with administrative goals. Our percentage rate for discharge orders written before 10:00 am was in the single digits, and some days it was zero. Our solution was to send a daily email to our providers that revealed each provider's percentage of discharges by 10:00 am. Everyone could see their rank. No financial incentive was attached to the metric at that time, just the knowledge of how each provider ranked among their peers. That was it: transparency! Our cumulative discharge before 10:00 am percentage increased to >50% in less than 3 months.

But we didn’t stop there. We decided to be transparent with all of our metrics. Now all of our hospitalist metric dashboards have complete transparency to all providers as well as administration. No secrets. The results? Our performance has skyrocketed in all metrics. Clinical documentation improvement inquiry response rates went to nearly 100%. Discharge summaries were all completed on time. Case mix index and CC/MCC/HCC (complication or comorbidity/major complication or comorbidity/hierarchical condition category) capture rates all improved. Our providers did not express embarrassment or hurt feelings. Collegiality increased, and many providers sought out individuals who were performing better for advice on how to improve their scores. The more successful providers freely offered advice and help to providers missing the mark on a metric. Our providers didn’t get embarrassed. They didn’t get hurt feelings. They thrived.

We now compare our providers’ cumulative scores to a benchmark derived from 10 other regional health systems of similar size and composition. Our scores and performance have become a team effort. Our team has risen to the challenge, and we have begun outperforming most of the other facilities in our comparison cohort. For our group, transparency didn’t manipulate behavior; transparency motivated it and improved performance.

We do employ financial incentives tied to performance, but the incentive accounts for only about 10% of the total income that is at risk. There is no doubt that the financial incentives work to some degree. But at least at our institution, in our hospitalist group, transparency is the major driver of performance.

Transparency in health care has become an expectation from consumers, insurance companies, and our government. We made it an expectation and an asset within our own health system. Perhaps transparency could play a new role in the success of your health system as well.
